# A single center experience of adjusting valve pressure ventriculoperitoneal shunts for the treatment of hydrocephalus in infants under 6 months old

**DOI:** 10.1371/journal.pone.0282571

**Published:** 2023-03-16

**Authors:** Adriano Cattani, Franziska Schwarzer, Mario Schwarzer, Andrea Spyrantis, Gerhard Marquardt, Susanne Schubert-Bast, Volker Seifert, Thomas M. Freiman

**Affiliations:** 1 Department of Neurosurgery, Goethe-University Hospital, Frankfurt am Main, Germany; 2 Department of Neuropaediatrics, Goethe-University Hospital, Frankfurt am Main, Germany; I.R.C.C.S. San Raffaele Scientific Institute, Vita-Salute San Raffaele University, ITALY

## Abstract

**Introduction:**

Ventriculoperitoneal shunt (VPS) with adjustable differential pressure valves are commonly used to treat infants with hydrocephalus avoiding shunt related under- or overdrainage. The aim of this study was to analyse the influence of VPS adjustable differential pressure valve on the head circumference (HC) and ventricular size (VS) stabilization in infants with post intraventricular haemorrhage, acquired and congenital hydrocephali.

**Methods:**

Forty-three hydrocephalic infants under 6 months old were prospectively included between 2014 and 2018. All patients were treated using a VPS with adjustable differential pressure valve. HC and transfontanelle ultrasonographic VS measurements were regularly performed and pressure valve modifications were done aiming HC and VS percentiles between the 25^th^ and 75^th^. The patients were divided into two groups: infants with hydrocephalus due to an intraventricular haemorrhage (IVH-H), and infants with hydrocephalus due to other aetiologies (OAE-H).

**Results:**

The mean of pressure valve modification was 3.7 per patient in the IVH-H group, versus 2.95 in the OAE-H group. The median of last pressure valve value was higher at 8.5 cm H_2_O in the IVH-H group comparing to 5 cm H_2_O in the OAE-H group (p = 0.013).

**Conclusion:**

Optimal VPS pressure valve values could be extremely difficult to settle in order to gain normalisation of the HC and VS in infants. However, after long term follow up (mean of 18 months) and several pressure valve modifications, this normalisation is possible and shows that infants with IVH-H need a higher pressure valve value comparing to infants with OAE-H.

## Introduction

The treatment of children hydrocephalus is one of the most common pediatric neurosurgery procedures and several techniques are used in order to establish a cerebral spinal fluid (CSF) dynamics balance in the brain. Generally it has to be distinguished between an occlusive and a communicating hydrocephalus with or without malabsorption. Since newborn infants are not often to be considered for endoscopic third ventriculostomy [1–7], VPS implantation is the treatment of choice for occlusive and malabsorptive hydrocephalus, despite its possible complications like infection, malpositioning and dysfunction with over- or underdrainage. The central part of the shunt system is the pressure valve. The optimal pressure valve value in hydrocephalic children remains unclear, however it seems to be fundamental to avoid under- or overdrainage [8–13]. VPS underdrainage can lead to ventriculomegaly, giant head circumference or blindness as well as overdrainage can lead to slit-ventricle syndrome, hyperostosis, chronic headaches and microcephaly [14–18]. To avoid VPS over- or underdrainage the selection and settings of the pressure valve is crucial [19–22]. In infants, unable to stand in upright position, a simple differential pressure valve might be sufficient. After the age of two years old, when the child is able to stand up, a gravitational unit addition might be mandatory [23, 24]. It has been shown, that the intracranial pressure rises with age. Also the different etiologies of hydrocephalus as hemorrhagic, infection, congenital, aquaeductal stenosis, Chiari malformation might have an influence on the settings of the pressure valve. Over the last years adjustable differential pressure valves have gained popularity over mono-pressure valves, both systems can be combined with a gravitational unit. The advantage of adjustable valves over mono-pressure valves is the possibility to adjust the pressure valve value as needed over the time. However, several questions remain unanswered concerning the advantages and disadvantages of using an adjustable differential pressure valve system in infants. Therefore, we have prospectively followed up every newborn child treated in our department with hydrocephalus due to different aetiologies performing adjustments on the implanted pressure valve according to the HC and transfontanelle ultrasonography VS avoiding under- or overdrainage and obtaining reliable HC percentile.

## Methods

This study was approved by the ethic committee Goethe-University Hospital, Frankfurt am Main which waved the need for parents or guardians consent. We included forty-three infants with hydrocephalus aged of one day to 6 months old in a prospective single-center study between 2014 and 2018. All patients were implanted with the Aesculap-Miethke-proGAV-shunt system, consisting of a ventricle and abdominal catheter, pumping-chamber reservoir, adjustable differential pressure valve ranging from 0 to 20 cm H_2_O and a fixed gravitational unit at 20 cm H_2_O. Indication for VPS insertion was crucially analysed taking into account several factors like signs of elevated intracerebral pressure, bradycardia, restlessness, shrill crying, vomiting as well as fast growing HC together with increased VS. In children with possible CSF access (e.g. with temporary CSF reservoir), analysis of CSF was performed to determined lower levels of protein and erythrocytes as well the absence of bacteria before VPS insertion to avoid VPS dysfunction or/and infection. Perioperative antibiotics were given following institutional standards. After surgery all patients received cranial and abdominal low dose radiographic control to exclude proximal and/or distal catheter misposition. At VPS implantation the standard pressure valve value was initially set to 5 cm H_2_O in almost all cases. Body weight, body height, HC and VS at birth as well at VPS insertion were documented. Measurements of HC and transfontanelle ultrasonography VS were prospectively performed with initial follow-up amounted at day 3, 5 and 7 after VPS implantation and continuously every 2 weeks in most of the cases. The end of the follow-up was determined once HC and VS percentile reached the range between 25^th^ and 75^th^ and stayed at this range during at least 3 months without any other additional pressure valve modification. Criteria for valve adjustments were based on significant drops or rises in the HC percentile and/or in VS. We used the HC growth curve of Fenton and collaborators for preterm children [25] and WHO Child Growth Standards for term born children (World Health Organization, 2009) to calculate HC percentile and Levene’s and/or Liao ventricle index curve to calculate VS percentile [26, 27]. We corrected the HC percentile as a function of weight and height ratio to avoid error due to stature specificity of each child. When the HC percentile figured between 95^th^ and 90^th^, the VPS valve pressure was 3 to 4 cm H_2_O down regulated and when between 90^th^ and 75^th^ down regulation of 2 cm H_2_O were performed. Similar was set for lower HC percentile and valve pressure was 3 to 4 cm H_2_O up regulated once HC percentile was between 5^th^ and 10^th^ and 2 cm H_2_O up regulated when HC percentile between 10^th^ and 25^th^. No modifications were done when HC percentile represented between 25^th^ and 75^th^. Only when VS was over 97^th^ or under 3^rd^ percentile, the valve pressure was carefully 2 cm H_2_O down or up regulated, respectively.The patients were divided into two groups: post intraventricular haemorrhage hydrocephalus (IVH-H; n = 20; 8 girls and 12 boys), and infants with congenital or hydrocephalus due to other aetiology (OAE-H; n = 23; 13 girls and 10 boys) and analysed separately. The software used for statistical analysis was Statistical Package for the Social Sciences (SPSS). All values are given in percentage, mean, standard deviation, confidence interval at 95% (CI_95_), median and interquartile range (IQR). The comparison between groups was performed using ANOVA and Mann-Whitney U test and p values < 0.05 were considered statistically significant.

## Results

### Demographic data of shunt-implanted children

The corrected gestational mean age of all 43 patients (21 girls and 22 boys) at the initial VPS insertion was 2.1 ± 2.2 months (CI_95_ 0.7). The mean follow-up was 18.9 ± 14.2 months (CI_95_ 4.3). The causes of hydrocephali were diverse. By far, the most frequent cause was due to intraventricular haemorrhage (IVH-H, 20 of 43 children, 46.5%, see Table 1).

**Table 1:** Demographic data of shunt-implanted children.

	IVH-H	OAE-H	All
Total	20 (46.5%)	23 (53.5%)	43
Sex			
Female	8	13	21
Male	12	10	22
Gestational age (weeks)			
Term (>37)	7	16	23
Preterm (28–37)	13	7	20
Extremely preterm (<28)	8	0	8
Very preterm (28–31)	4	1	5
Moderately preterm (32–33)	1	1	2
Late preterm (34–37)	0	5	5
Mean weight at birth (grams)	1704.2 ± 1089.3	2919.1 ± 638.7	2354 ± 1061.3
Mean height at birth (cm)	39.4 ± 7.5	48.5 ± 3.9	44.3 ± 7.3
Mean head circumference at birth (cm)	28.6 ± 5.4	35.5 ± 3.9	32.3 ± 5.8
Temporary reservoir before shunt insertion	16	4	20
Mean age at initial shunt insertion (months)	2.2 ± 1.5	1.9 ± 2.8	2.1 ± 2.2
Mean weight at shunt insertion (grams)	3287 ± 1142	4209.3 ± 1688	3780.3 ± 1516
Mean height at shunt insertion (cm)	48.9 ± 7.7	53.7 ± 7.5	51.5 ± 7.9
Mean head circumference at shunt insertion (cm)	34.8 ± 3.8	39.5 ± 4.7	37.4 ± 4.9
Mean children follow-up (months)	21.6 ± 16	16.6 ± 12.3	18.9 ± 14.2
Cause of hydrocephalus			
Intraventricular haemorrhage	20	1*	21
Aquaeductal stenosis		5	5
Brain infection		4	4
Myelomeningocelia		2	2
Dandy Walker maformation		2	2
Pfeiffer syndrom		1	1
Arnold-Chiari malformation type II		1	1
Meningocelia		1	1
Malabsorptive hydrocephalus		1	1
Occlusive hydrocephalus		3	3
Spina bifida		2	2

All values are expressed in percentage, mean and standard deviation.

*Intraventricular haemorrhage secondary to brain infection; IVH-H: children with post intraventricular haemorrhage hydrocephalus; OAE-H: children with hydrocephalus due to other aetiology.

Hydrocephalus due to other aetiology (OAE-H) are described below and were grouped together to enable better assessment of the effectiveness of VPS therapy in managing children hydrocephalus. Among the 43 patients, 20 (8 girls and 12 boys; 46.5%) suffered from hydrocephalus after intraventricular haemorrhage (IVH-H) and 23 (10 boys and 13 girls; 53.5%) due to different aetiologies (OAE-H) including aquaeductal stenosis (n = 5), brain infection (n = 4), myelomeningocelia (n = 2), meningocelia (n = 1), spina bifida (n = 2), Dandy-Walker malformation (n = 2), Arnold-Chiari malformation type II (n = 1), Pfeiffer syndrom (n = 1), occlusive hydrocephalus (n = 3), malabsorptive hydrocephalus (n = 1) and one child developed hydrocephalus after severe intracerebral haemorrhage due to a brain infection (Table 1). As to be expected, the rate of preterm children was higher in the IVH-H group (13 out of 20; 65%) including extremely preterm (< 1500g, 8 out of 20; 40%) compared to the OAE-H group (7 out of 23; ~30%) and the mean weight at birth was 1704.2 ± 1089.3 grams (CI_95_ 509.8) and 2919.1 ± 638.7 grams (CI_95_ 276.2), respectively. The mean height at birth was 39.4 ± 7.5 centimetres (CI_95_ 3.5) in the IVH-H infants and 48.5 ± 3.9 centimetres (CI_95_ 1.7) in the OAE-H infants. All together the mean weight at birth of all 43 patients was 2354 ± 1061.3 grams (CI_95_ 326.6) and the mean height at birth 44.3 ± 7.3 centimetres (CI_95_ 2.3). The mean of head circumference in the IVH-H group was 28.6 ± 5.4 centimetres (CI_95_ 2.5) compared to 35.5 ± 3.9 centimetres (CI_95_ 1.7) in the OAE-H group and all together among the 43 patients 32.3 ± 5.8 centimetres (CI_95_ 1.8).

Eighty percent of all IVH-H patients (16 out of 20) were initially treated through external reservoir before definitive VPS insertion and only 17% approximately (4 out of 23) in the OAE-H group. The mean age at VPS insertion was 2.2 ± 1.5 months (CI_95_ 0.7) in the IVH-H children versus 1.9 ± 2.8 months (CI_95_ 1.2) in the OAE-H children. The mean weight at VPS insertion was 3287 ± 1142 grams (CI_95_ 534.3) for infants with IVH-H and 4209.3 ± 1688 grams (CI_95_ 730) for infants with OAE-H. The mean height at VPS insertion was 48.9 ± 7.7 centimetres (CI_95_ 3.6) and 53.7 ± 7.5 centimetres (CI_95_ 3.3), respectively. All together, at VPS insertion of all 43 patients, the mean weight was 3780.3 ± 1516 grams (CI_95_ 467) and the mean height was 51.5 ± 7.9 centimetres (CI_95_ 2.4). The mean HC for IVH-H infants was 34.8 ± 3.8 centimetres (CI_95_ 1.8) and 39.5 ± 4.7 centimetres (CI_95_ 2) for OAE-H and all together between all 43 patients was 37.4 ± 4.9 centimetres (CI_95_ 1.5). The mean follow-up was 21.6 ± 16 months (CI_95_ 7.5) for IVH-H infants versus 16.6 ± 12.3 months (CI_95_ 5.3) for OAE-H infants (Table 1).

### Valve pressure regulation during prospective follow up after shunt insertion

The standard valve pressure value was initially set at 5 cm H_2_O, except if the VS was very large, which was the case in 2 children with IVH-H and in 5 children with OAE-H and therefore the valve pressure values were set higher as 5 cm H_2_O to avoid overdrainage. Pressure valve values lower than 5 cm H_2_O were not used at initially VPS insertion. Among all 43 patients, the mean pressure valve value at initial VPS insertion was 5.79 ± 2.05 cm H_2_O (CI_95_ 0.63; median IQR 5 (5–5)). The initial mean pressure valve value was 5.4 ± 1.27 cm H_2_O (CI_95_ 0.59) with median and IQR of 5 (5–5) for the IVH-H group and 6.13 ± 2.53 cm H_2_O (CI_95_ 1.09) also with median and IQR of 5 (5–5) for the OAE-H group (Table 2). In the course of our patients’ follow-up, we aimed to reach HC and VS percentiles between 25^th^ and 50^th^. To this issue 70% of the children with IVH-H and 61% of the children with OAE-H required more than one pressure valve value modification. The mean number of pressure valve modifications was 3.7 ± 3.04 (CI_95_ 1.42) with median and IQR of 2.5 (1–5.25) and 2.95 ± 3.28 (CI_95_ 1.41) with median and IQR of 2 (1–4), respectively. All together there was a mean of valve pressure modifications from 3.3 ± 3.15 (CI_95_ 0.97) with median and IQR of 2 (1–5) in all 43 patients (Table 2).

**Table 2:** Amount and direction of valve pressure regulation.

	IVH-H	OAE-H	All
Valve pressure at shunt insertion (cm H_2_O)			
Mean value	5.40±1.27	6.13±2.53	5.79±2.05
Median value	5 (5–5)	5 (5–5)	5 (5–5)
Rate of valve pressure-modification requirement			
0 modifications	10%	22%	16%
1 modification	20%	17%	19%
> 1 modification	70%	61%	65%
Number of valve pressure modifications			
Mean value	3.7±3.04	2.95±3.28	3.3±3.15
Median value	2.5 (1–5.25)	2 (1–4)	2 (1–5)
Up-regulation after adjustments			
Rate	65%	35%	49%
Mean value (cm H_2_O)	9.76±2.89	8.5±1.19	9.28±2.43
Median value (cm H_2_O)	10 (8–10)	8.5 (7.75–9.25)	9 (8–10)
Down-regulation after adjustments			
Rate	20%	35%	28%
Mean value (cm H_2_O)	5±2.82	2.62±2.26	3.41±2.61
Median value (cm H_2_O)	5 (4–6)	4 (0–5)	3.5 (2.25–5)
Same valve pressure after adjustments			
Rate	15%	30%	23%
Mean value (cm H_2_O)	5±0	5.57±1.51	5.4±1.26
Median value (cm H_2_O)	5 (5–5)	5 (5–5)	5 (5–5)
Valve pressure at the end of follow-up*			
Mean value (cm H_2_O)	8.1 ± 3.46	5.56 ± 2.99	6.74 ± 3.43
Median value (cm H_2_O)	8.5 (5–10)	5 (4.5–8)	7 (5–9)

All values are expressed in percentage, mean, standard deviation, median and interquartile range (IQR). IVH-H: children with post intraventricular haemorrhage hydrocephalus; OAE-H: children with hydrocephalus due to other aetiology; *The comparison of the mean and median of valve pressure at the end of follow-up between both groups was statistically significant (Anova p = 0.013 and Mann-Whitney U test p = 0.022, respectively).

**Fig 1:**
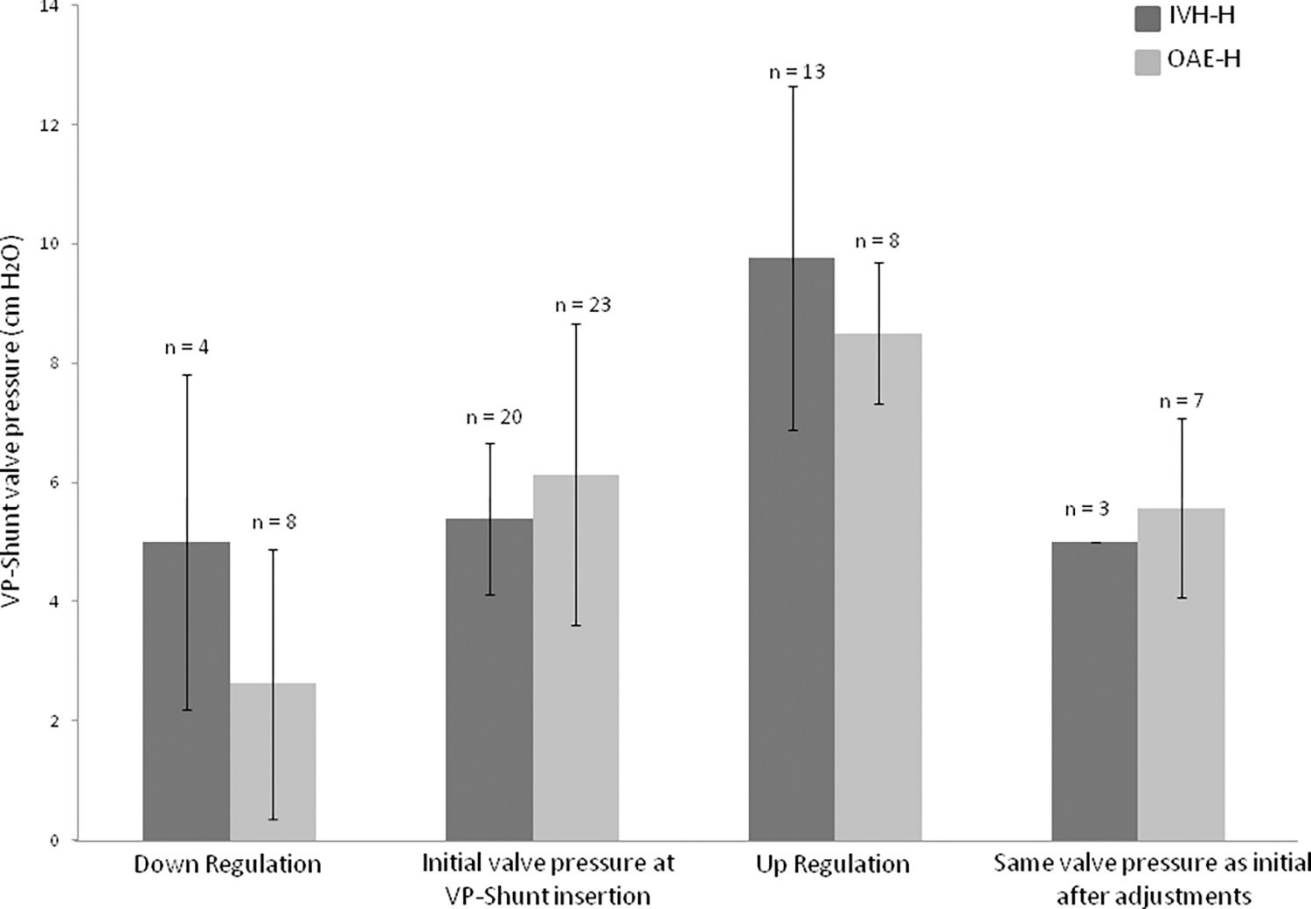
Summary of up and down valve pressure regulations and mean valve pressure values with standard deviation. In dark gray the IVH-H group (n = 20) showing a mean of 5.40 ± 1.27 cm H_2_O valve pressure at VPS insertion; in 4 patient the valve pressure was down regulated (mean of 5 ± 2.82 cm H_2_O) and in 13 patients it was up regulated (mean of 9.76 ± 2.89 cm H_2_O); in 3 patients the valve pressure was settled back to initial value after modifications. In light gray the OAE-H group (n = 23) showing a mean of 6.13 ± 2.53 cm H_2_O valve pressure at VPS insertion; in 8 patients the valve pressure was down regulated (mean of 2.62 ± 2.26 cm H_2_O) and in 8 patients it was up regulated (mean of 8.5 ± 1.19 cm H_2_O); in 7 patients the valve pressure was settled back to initial value after modifications (IVH-H: children with post intraventricular haemorrhage hydrocephalus; OAE-H: children with hydrocephalus due to other aetiology; VPS: ventriculoperitoneal shunt).

The valve pressure was up regulated in 65% of the children with IVH-H with a mean value of 9.76 ± 2.89 cm H_2_O (CI_95_ 1.74) and with median and IQR of 10 (8–10) and in 35% of the children with OAE-H with a mean value of 8.5 ± 1.19 cm H_2_O (CI_95_ 0.99) and with median and IQR of 8.5 (7.75–9.25). In all 43 patients 49% valve pressures had to be up regulated with a mean of 9.28 ± 2.43 cm H_2_O (CI_95_ 1.1) and with median and IQR of 9 (8–10). In the other hand, down regulation was performed in only 20% of IVH-H patients with mean value of 5 ± 2.82 cm H_2_O (CI_95_ 4.5) and with median and IQR of 5 (4–6) and in 35% of the OAE-H patients with mean value of 2.62 ± 2.26 cm H_2_O (CI_95_ 1.89) and with median and IQR of 4 (0–5). The rate of down regulation in all 43 patients was 28% with mean value of 3.41 ± 2.61 cm H_2_O (CI_95_ 1.65) and with median and IQR of 3.5 (2.25–5). For 15% and 30%, respectively with a total of 23% for all 43 patients, the same initial valve pressure value was settled after modifications (Table 2 and Fig 1).

Finally, the mean of last and optimal pressure valve value after modifications amounted up to 8.1 ± 3.46 cm H_2_O (CI_95_ 1.62) with median and IQR of 8.5 (5–10) in the IVH-H group versus 5.56 ± 2.99 cm H_2_O (CI_95_ 1.29) with median and IQR of 5 (4.5–8) in the OAE-H group. This difference was statically significant (Anova for mean with p = 0.013 and Mann-Whitney U test for median with p = 0,022). The mean of last valve pressure value, including all 43 patients, was 6.74 ± 3.43 cm H_2_O (CI_95_ 1.05) with median and IQR of 7 (5–9) (Table 2 and S1 Fig).

## Discussion

The main findings of this study were that 84% of children with hydrocephalus treated with a VPS system with an adjustable pressure valve required pressure modification. Ninety percent of the infants with hydrocephalus due to intraventricular haemorrhage (IVH-H) needed pressure valve modifications comparing to 78% of the infants with hydrocephalus due to other aetiologies (OAE-H). The last and optimal median pressure valve value for which the patients presented a stable HC percentile was 8.5 cm H_2_O (mean 8.1 cm H_2_O) in the IVH-H group and 5 cm H_2_O (mean 5.6 cm H_2_O) in the OAE-H group.

**Fig 2:**
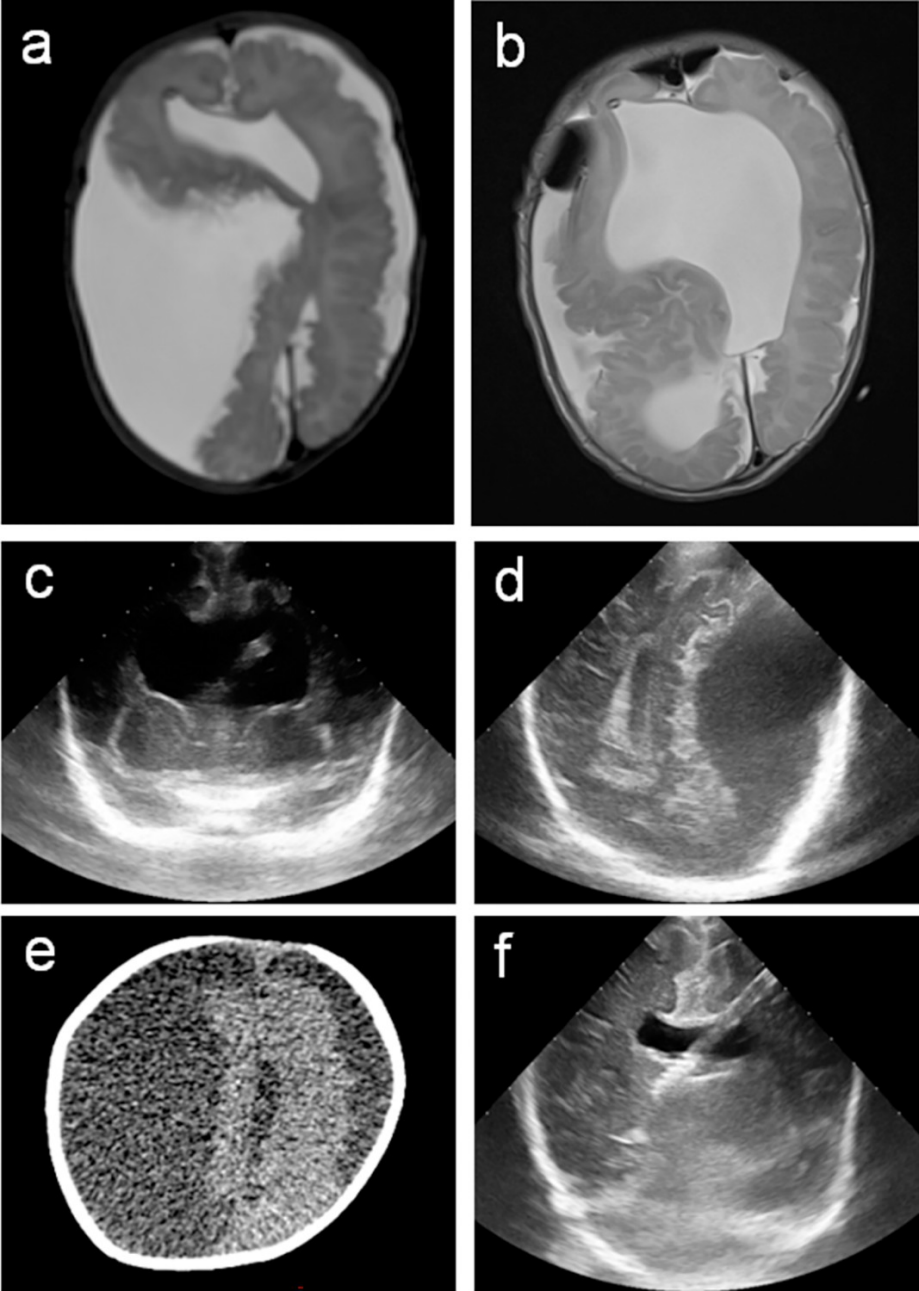
Complications due to over drainage after VPS implantation. T2 sequence magnetic resonance imaging control one month after VPS implantation in a 35 days old newborn with aquaeductal stenosis and hydrocephalus (a and b). a; Bilateral hygroma complication with midline shift due to overdrainage (valve pressure at 5 cm H_2_0). The patient had no clinical symptoms correlation. b; Post operative control imaging showing hygroma regression. The valve pressure was changed intra operatively to 8 cm H_2_O. c; Transfontanelle ultrasonography control right after VPS implantation in a 50 days old newborn with aquaeductal stenosis and hydrocephalus. The intra ventricular catheter is identified with correct position in the right ventricle and the valve pressure was initially settled at 5 cm H_2_0. d; Two weeks later at regular visit transfontanelle ultrasonography control showing bilateral hygroma. The patient presented no clinical symptoms. e; pre operative low dosis computer tomography control confirming the diagnosis. f; Transfontanelle ultrasonography control 3 days after hygroma evacuation and valve pressure correction to 8 cm H_2_O (VPS: ventriculoperitoneal shunt).

Changing the valve pressure value was made according to the HC and/or transfontanelle ultrasonography VS measurements. Most of the cases, HC measurement was a good indicator of under- and overdrainage during follow-up suggesting the required direction of valve pressure adjustment. However, we observed two cases of overdrainage leading to bilateral hygroma in asymptomatic children with stabilised HC percentile (Fig 2).

**Fig 3:**
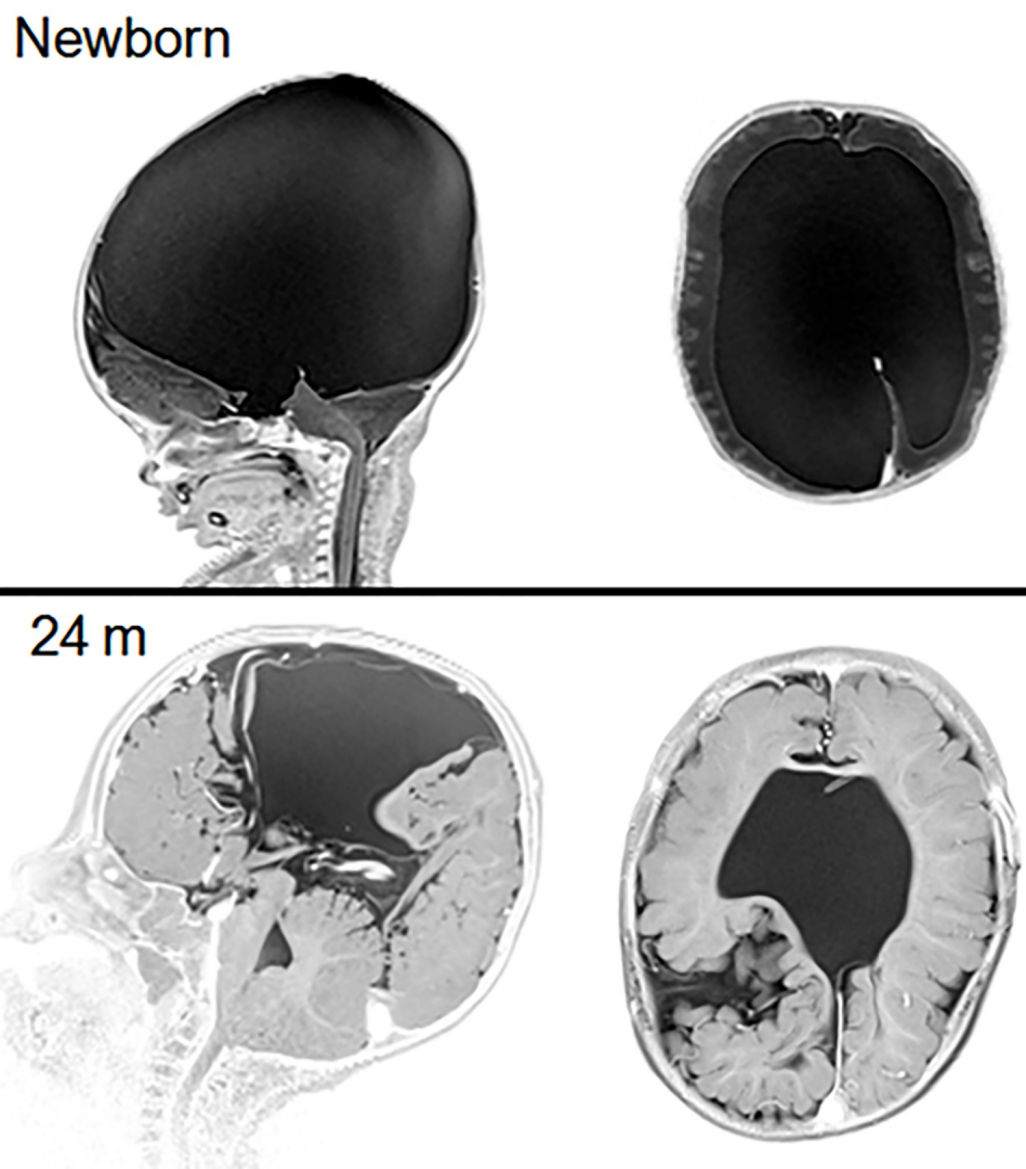
Representative example of boy with hydrocephalus due to aquaeductal stenosis. On the top, T2 sequence magnetic resonance imaging at birth showing the aquaeductal stenosis and the consequent hydrocephalus. On the bottom, T2 sequence magnetic resonance imaging control at 2 years old showing excellent cortical growth and normalization of the HC after VPS implantation. The pressure valve needed to be modified 8 times and the final pressure valve value amounted to 3 cm H_2_O (VPS: ventriculoperitoneal shunt; HC: head circumference).

This observation means that overdrainage is not necessarily accompanied with changes in HC measurements toward smaller value reinforcing the important role of transfontanelle cerebral ultrasonography. Therefore we recommend systematic follow-up period of at least 2 to 3 months after shunt insertion, even in absence of symptoms. Finally, the relatively small size and monocentric origin of patients sample as well as the follow up period have to be considered before making definitive conclusions. Despite these limitations, we believe that these results indicate that using an adjustable differential pressure valve presents the advantage of adjusting pressure as needed to efficiently manage hydrocephalus in children regardless aetiologies (see Fig 3 for a case illustration).

Inserting an adjustable differential pressure valve and a gravitational unit at a later time point decrease the risk of shunt infection and other complications in childhood [22, 28–30]. However, there are no standards regarding the usage of these valves or their optimal timing of insertion in newborn, in particular in preterm children. Indeed, due to the large-size of those adjustable differential pressure valves and to the possibility of valve obstructions because of complexity of valve design, some institutions prefer to use smaller valves with fixed pressure only instead of adjustable differential pressure valve with or without gravitational unit [11]. Concerning the gravitational unit, it has been shown its efficacy in avoiding overdrainage and slit-ventricle syndrome and could be considered an essential component of a VPS system [9, 10, 14, 31]. In our study we did not observe any complication due to the size of proGAV system including the gravitational unit in children below walking age. Therefore, according to our high rate of pressure valve adjustments mentioned above, we justified here the use of an adjustable differential pressure valve system in order to avoid possible complications caused by additional surgery in case of changing the mono-pressure valves or inserting a gravitational units to adapt the amount of CSF diversion according to each patient reaching normal age-related percentile. Others authors also mentioned no significant complication rate using adjustable differential pressure valve to treat paediatric hydrocephalus [11, 15, 18, 32, 33].

**Fig 4:**
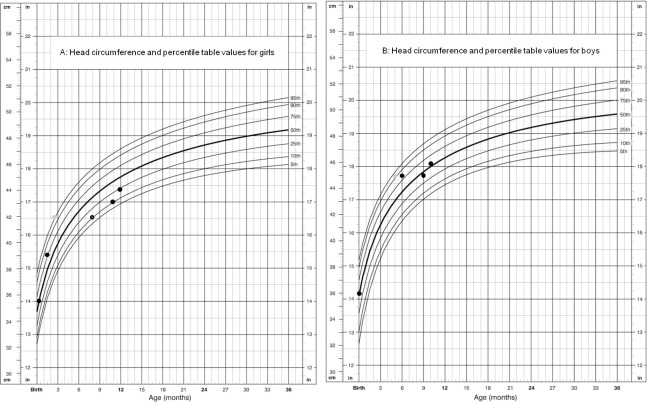
A. The HC stabilisation after VPS Implantation in a girl with IVH-H. At birth the child presented HC of 35.5 cm (50th Percentile; black dots). At the age of 1.5 months the HC raised to 39 cm (75th Percentile). One month later the HC increased to 42 cm showing too fast and abnormal acceleration of HC. VPS was then implanted with initial valve pressure of 5 cm H_2_O (gray dot). The HC and transfontanelle ultrasonography VS measurements were performed every 2 weeks and the HC stabilized at 42 cm during 5.5 months. Due to this small and persistent HC the valve pressure was adjusted to 7 cm H_2_O (white center black dot) and at the 11th and 12th months HC normal growth stabilization was achieved (VPS: ventriculoperitoneal shunt; HC: head circumference; VS: ventricular size). B. The HC stabilization after VPS Implantation in a boy with hydrocephalus due to aquaeductal stenosis. At birth the child presented HC of 36 cm (50th Percentile; black dots). At the age of 5 months the HC raised to 45 cm (90th Percentile) and the diagnosis was established through magnetic resonance imaging. In order to avoid a drastic macrocephaly a VPS with initial valve pressure of 5 cm H_2_O was implanted (gray dot). The HC and transfontanelle ultrasonography VS measurements were performed every 2 weeks. One month later the head stopped growing and a complete stabilization of the HC was achieved without valve adjustments between the 9th and 10th months (VPS: ventriculoperitoneal shunt; HC: head circumference; VS: ventricular size; Growth Charts adapted from Centers for Disease Control and Prevention United States; May 30, 2000).

The explanation of different last and optimal pressure valve values between both groups in this study remains speculative. However, it might very likely that after intraventricular haemorrhage, the infants develop CSF malabsorption due to CSF containing blood degradation proteins [34–36]. Therefore, infants with IVH-H not only need to wait for decent body weight and height, but also they have to be pre-treated temporaly using ventricular catheter reservoir, sub-galeal shunts or lumbar puncture until lower levels of CSF proteins before VPS insertion [37]. Those additional manipulations might increase the complication rate such as parenchymal damage as well as porencephaly and infection [28, 38–41]. On the other hand, hydrocephalus due to other aetiologies, in particular congenital lesions as aquaeductal stenosis and brain malformation, is characterised by obstruction of CSF flow leading to chronicle disturbances of CSF dynamics already in utero. Accordingly, CSF malabsorption in the IVH-H group could be better regulated with a higher opening pressure of the shunt valve, whereas CSF obstruction in the OAE-H group could be better regulated with lower opening pressure to achieve normal HC and VS (see Fig 4A and 4B for illustration).

Up to date in the literature, there is no clear explanation about those pathomechanisms and more studies are needed to better understand different hydrocephalus dynamics.

## Supporting information

S1 Fig Time-Plot illustration summarizing valve pressure adjustments. Black dotted lines represent IVH-H group and gray continuous lines represent OAE-H. Note the black and gray bold lines show the mean of optimal pressure valve value for IVH-H (8.1 cm H2O) and OAE-H (5.56 cm H2O), respectively at the end of follow up.
(TIF)

S1 Data (XLSX)
