# Supplementary figures and images for: A single center experience of adjusting valve pressure ventriculoperitoneal shunts for the treatment of hydrocephalus in infants under 6 months old
Source: PLoS One. 2023 Mar 16;18(3):e0282571. doi: 10.1371/journal.pone.0282571 (PMC10019726; doi:10.1371/journal.pone.0282571)

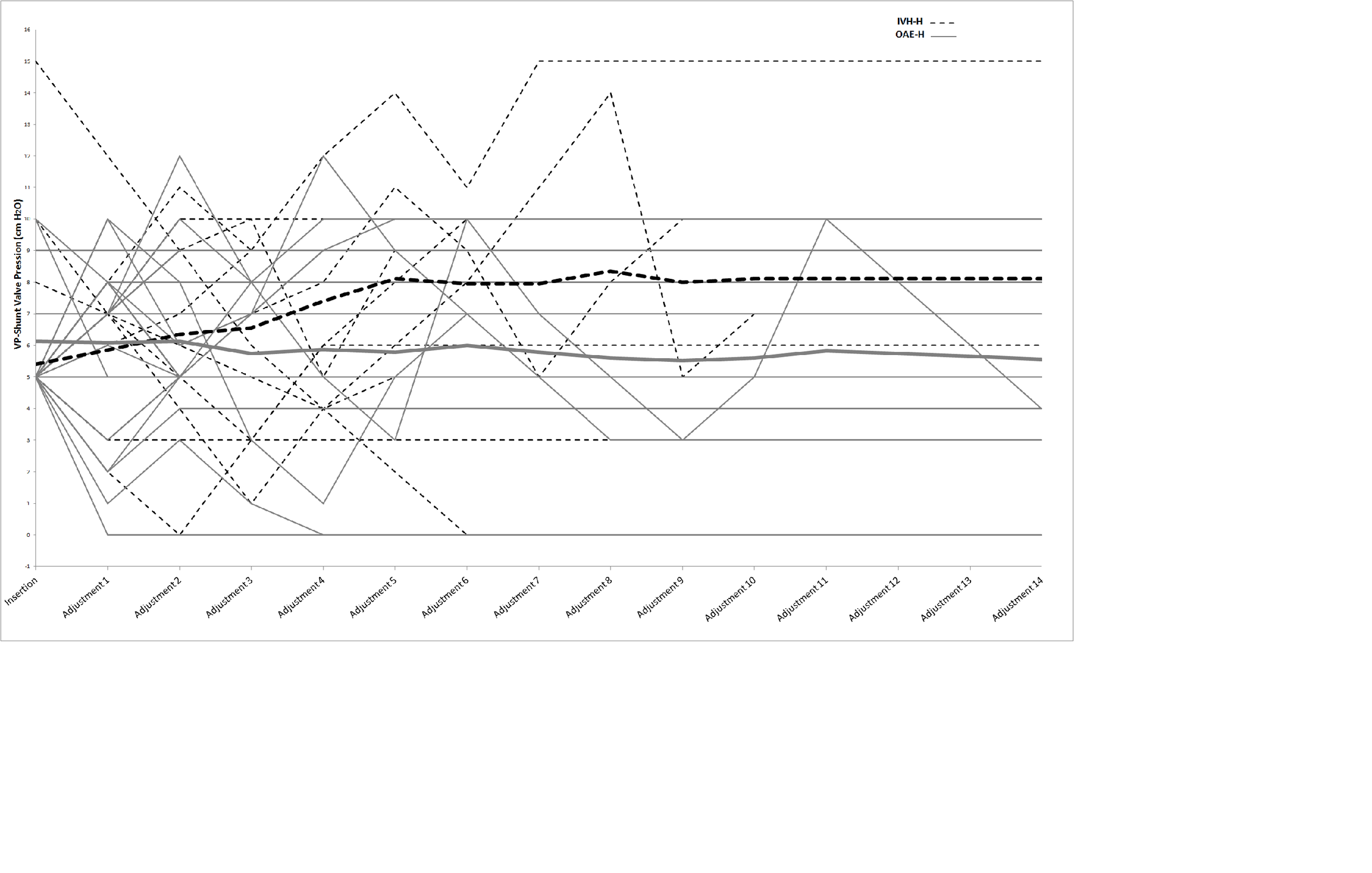

Supplement: S1 Fig — Black dotted lines represent IVH-H group and gray continuous lines represent OAE-H. Note the black and gray bold lines show the mean of optimal pressure valve value for IVH-H (8.1 cm H2O) and OAE-H (5.56 cm H2O), respectively at the end of follow up. (TIF) [file pone.0282571.s001.tif]
